# Peripheral biomarkers of treatment-resistant schizophrenia: Genetic, inflammation and stress perspectives

**DOI:** 10.3389/fphar.2022.1005702

**Published:** 2022-10-12

**Authors:** Shimeng Jiao, Ting Cao, Hualin Cai

**Affiliations:** ^1^ Department of Pharmacy, The Second Xiangya Hospital, Central South University, Changsha, China; ^2^ Institute of Clinical Pharmacy, Central South University, Changsha, China; ^3^ International Research Center for Precision Medicine, Transformative Technology and Software Services, Changsha, Hunan, China

**Keywords:** treatment resistant schizophrenia (TRS), gene, inflammation, stress, neurobiology, peripheral biomarkers

## Abstract

Treatment-resistant schizophrenia (TRS) often results in severe disability and functional impairment. Currently, the diagnosis of TRS is largely exclusionary and emphasizes the improvement of symptoms that may not be detected early and treated according to TRS guideline. As the gold standard, clozapine is the most prescribed selection for TRS. Therefore, how to predict TRS in advance is critical for forming subsequent treatment strategy especially clozapine is used during the early stage of TRS. Although mounting studies have identified certain clinical factors and neuroimaging characteristics associated with treatment response in schizophrenia, the predictors for TRS remain to be explored. Biomarkers, particularly for peripheral biomarkers, show great potential in predicting TRS in view of their predictive validity, noninvasiveness, ease of testing and low cost that would enable their widespread use. Recent evidence supports that the pathogenesis of TRS may be involved in abnormal neurotransmitter systems, inflammation and stress. Due to the heterogeneity of TRS and the lack of consensus in diagnostic criteria, it is difficult to compare extensive results among different studies. Based on the reported neurobiological mechanisms that may be associated with TRS, this paper narratively reviews the updates of peripheral biomarkers of TRS, from genetic and other related perspectives. Although current evidence regarding biomarkers in TRS remains fragmentary, when taken together, it can help to better understand the neurobiological interface of clinical phenotypes and psychiatric symptoms, which will enable individualized prediction and therapy for TRS in the long run.

## 1 Introduction

Schizophrenia is a severe, lifelong condition characterized by poor behavioral, emotional, and cognitive function that affects about 1% of the world’s population (Marder and Cannon, 2019). With the development of antipsychotic drugs in the 1950s, patients with schizophrenia were given the opportunity to be treated and rehabilitated (Nucifora et al., 2019). Unfortunately, not all patients respond to antipsychotic drugs, and around a third of patients were considered treatment-resistant after failing to respond to two different antipsychotic drugs (Faden and Citrome, 2019). Compared with schizophrenia in remission, the treatment cost for TRS is 3-11 fold higher, accounting for 60%–80% of the entire cost of schizophrenia management, which places a heavy financial burden on families and society (Kennedy et al., 2014). Patients who are resistant to treatment have much severer symptoms, disabilities, higher risk of suicide, lower quality of life as well as worse prognosis than patients who are responsive to treatment (Correll and Howes, 2021).

The definition of TRS has been continuously updated since 1966 (Itil et al., 1966), and the clinical focus on TRS switches from the persistence of positive symptoms to a stronger emphasis on comprehensive assessment of psychosocial functioning. The available standards for understanding and defining TRS have been illustrated in several reviews (Vita et al., 2019; Correll and Howes, 2021; Howes et al., 2022). However, the existent standards vary with studies, especially in the recommendations of optimum drug therapy and the methods for assessment of treatment response, which limited their research and clinical transformation, as well as restricted the formation of consistent clinical management (Howes et al., 2022). In order to address this issue, the Treatment Response and Resistance in Psychosis (TRRIP) Working Group has updated the consensus standards and guidelines based on the assessment of previously defined approaches (Howes et al., 2017). The term “treatment-resistant schizophrenia (TRS)” has been proposed to this category of schizophrenia patients who match the following characteristics: 1) at least two different antipsychotic drugs were used, 2) prescribed dose was the target dose for acute phase of schizophrenia or a total daily dose equivalent to 600 mg chlorpromazine, 3) each antipsychotic treatment algorism lasted at least 6 weeks and required at least 12 weeks of treatment time and 4) during the prospective observational period, the symptom improvement was less than 20% and the severity still reached the treatment-resistant threshold (moderate severity or above according to the standardized rating scale).

The only antipsychotic drug that has been proven to be effective in TRS is clozapine (Rajagopal et al., 2018). However, it is only effective in 40%–70% of patients with TRS (Samanaite et al., 2018). These patients who are resistant to clozapine were called ultra–treatment resistant schizophrenia (Howes et al., 2017). Patients who have not reacted to previous antipsychotic medications can benefit from clozapine, but it carries a risk of major side effects and demands frequent blood testing (Samanaite et al., 2018). For these reasons, clozapine treatment may be difficult for both patients and doctors. Therefore, there is a significant delay in starting clozapine treatment (mean delay 2–5 years) in regular clinical practice (Yoshimura et al., 2017). However, clozapine should be used for TRS patients especially during the early stage according to research (Horsdal et al., 2017; Egerton et al., 2021). Biomarkers of TRS can forecast the appearance of TRS and offer proof for clinicians to recommend clozapine. Additionally, if tests could be created to assist doctors in anticipating whether or not a patient will respond to clozapine, this would significantly shorten the time before clozapine is used and allow for selective use of clozapine in the subset of patients where it is most likely to be effective.

Currently, several demographic and clinical factors have been identified to predict worse response to treatment for schizophrenia, such as family history of psychosis, earlier age of onset, poor social functioning prior to onset, longer untreated time, more severe negative symptoms, and extrapyramidal symptoms shown early in treatment (Nucifora et al., 2019; Takeuchi et al., 2019; Correll and Howes, 2021; Cai et al., 2022). As for the neurobiological mechanisms underlying TRS, several hypotheses are proposed including dopamine hypersensitivity, high dopaminergic and normal dopaminergic subtypes, glutamate/GABAergic disorders, serotonin disorders, inflammation and stress (Potkin et al., 2020). However, the majority of available studies focused on how the preset treatment strategy improves psychiatric symptoms rather than on exploring the underlying neurobiological alterations, the biological mechanisms of the above mentioned risk factors (Bumb et al., 2015). As evidenced by the variability of antipsychotic treatment responsiveness, it has come to a consensus that TRS actually represents a distinct biological category with its own pathophysiology (Iasevoli et al., 2016; Nucifora et al., 2019). On one hand, the three elements, including a standard diagnosis of schizophrenia, antipsychotic drug use and duration of treatment, need to be present at the same time according to the most recent consensus standards defining TRS. However, several factors, such as poor patient compliance or a protracted patient response time, can influence this procedure. In addition, patients who developed TRS only after the initial response to antipsychotic medications fell into this category, making it impossible to diagnose TRS at the beginning of the first antipsychotic treatment (Howes et al., 2017; Howes et al., 2022). On the other hand, an accurate prediction of poor response to the treatment of schizophrenia based on clinical factors requires profound experiences and skills of the psychiatrists, and will also be affected by the subjective attitude of patients (Bumb et al., 2015). In this context, there is an increasing clinical need to predict the development of TRS with novel biomarkers.

The neuroimaging technology of TRS is relatively mature. It potentially serves as a tool for distinguishing TRS from non-TRS depending on brain structural and functional abnormalities. Most studies reported cerebral structure abnormalities include atrophied brain, enlarged ventricular, reduced cortical thickness, compromised integrity of gray matter and white matter (Vita et al., 2019). TRS showed a wide range of brain activity patterns and connectivity abnormalities, particularly in the frontotemporal and subcortical networks (Molent et al., 2019). The state-of-the-art neuroimaging technologies such as magnetic resonance spectroscopy (MRS or MRI), Computed Tomography (CT), functional Magnetic Resonance Imaging (fMRI), Positron Emission Tomography (PET), and Single Photon Emission Computed Tomography (SPECT) usually require professionals and are often associated with a high cost (Samanaite et al., 2018; Vita et al., 2019; Cao et al., 2020), despite the fact that they are noninvasive.

A biomarker is a biological, genetic, epigenetic, or chemical trait that can be used to diagnose, monitor, and predict disease (Kraguljac et al., 2021). Compared with neuroimaging biomarkers from central nervous system, peripheral biomarkers are more widely available, and cost-effective. Although the molecular causes and pathways of TRS are still unclear, accumulating studies are focusing on the exploration of validated, selective, and specific peripheral biomarkers for TRS in the hope of future routine use (Nedic Erjavec et al., 2018). This paper mainly reviews the scientific development of peripheral genetic, inflammation and stress biomarkers related to TRS, based on the neurobiological hypotheses currently implicated in TRS. The goal of this review is to provide new insights in monitoring and diagnosing TRS, which will allow for earlier intervention and noticing of a change in the disease’s course and severity (Nucifora et al., 2019). When taken together the data of peripheral TRS biomarkers, it will help us better understand the underlying neurological mechanisms of TRS and guide the development of future treatments (Schizophrenia Working Group of the Psychiatric Genomics Consortium, 2014).

## 2 Methods

**FIGURE 1:**
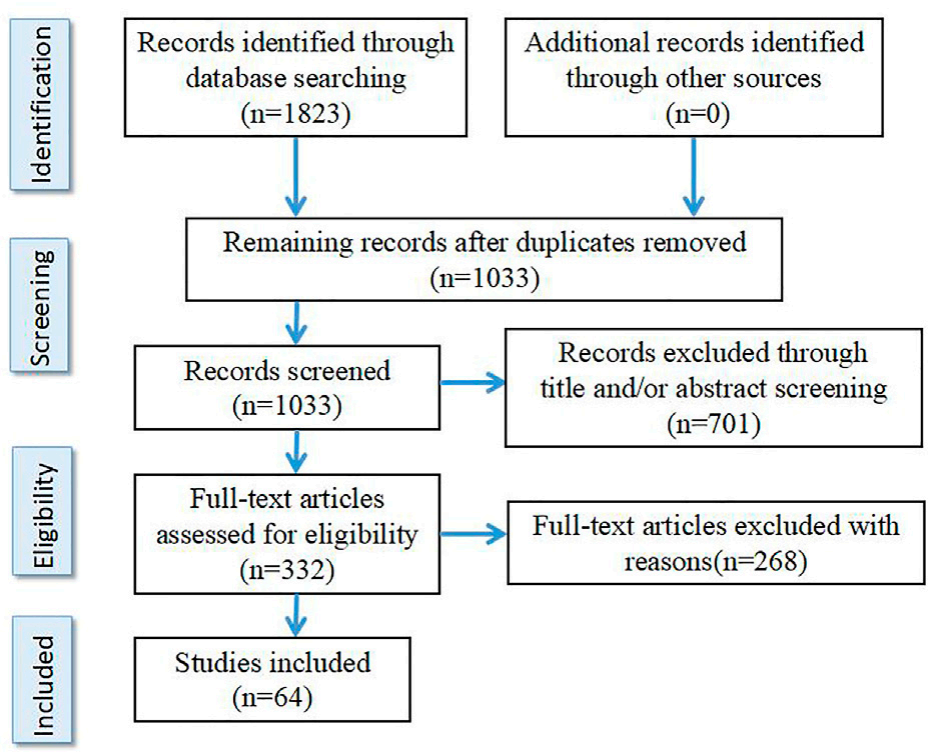
Flowchart of study selection.

The literature search was performed at the PubMed, Embase, BIOSIS and PsychInfo databases using the following keywords “schizophrenia,” “resistant,” “refractory,” “response,” “treatment,” “clozapine,” “biomarkers” and “peripheral biomarkers” as well as different combinations of these keywords. References are papers published before May 2022 including Clinical Trial, Meta-analysis, Randomized Controlled Trial and Review. The search results were deduplicated and screened. According to the inclusion criteria and exclusion criteria, two authors S.J. and T.C. independently screened the retrieved literature, and discussed together when the results were inconsistent. Selection criteria are as follows: 1) It is an original English paper. 2) The results are from clinical studies. 3) Therapeutic resistant schizophrenia was included only when clearly defined in the study according to criteria customized at the time. Exclusion criteria were: 1) The inclusion criteria for TRS were not clearly defined. 2) Studies on animals. In total, 64 studies were selected for this review (see Figure 1 for details).

## 3 Peripheral biomarkers

### 3.1 Genetic biomarkers

High heritability for schizophrenia emphasizes the important role of genetic variation in the etiology of this devastating disease (Legge et al., 2021). TRS may be even more heritable, with more significantly genetic underpinnings (Reynolds et al., 2006; Gillespie et al., 2017). Due to the variations in the effectiveness of antipsychotic drugs on controlling symptoms and inducing adverse side effects, antipsychotic pharmacogenetics issues have received considerable attention in recent years. The researchers exerted their energy primarily on identifying candidate genes that have been associated with the pharmacological actions and mechanisms of these antipsychotic drug receptors (Reynolds, 2004; Reynolds et al., 2006). A recent study utilized two large and orthogonally created databases to collate drug targets into 167 gene sets targeted by pharmacologically similar antipsychotic drugs, and then examined the enrichment of schizophrenia risk loci in these sets. Interestingly, the findings support the notion that there are specific genetic overlaps between schizophrenia pathogenesis and antipsychotic mechanism of action (Ruderfer et al., 2016). To identify genes with a role in both the risks of developing schizophrenia and treatment resistance, they tested gene sets that were previously enriched for the singleton disruptive mutations in schizophrenia, gene sets implicated in antipsychotic efficacy by PharmGKB and genes with known roles in ADME (absorption, distribution, metabolism, excretion) (Dahl, 2002; Purcell et al., 2014). The results indicated that the genes related to the targets of antipsychotic drugs are the only overlapped set showing enrichment for both disease risk and treatment resistance (Ruderfer et al., 2016).

Pharmacogenetic biomarkers from peripheral blood leucocytes can predict TRS through genetic variation by analyzing the correlation between the risk genes, namely single nucleotide polymorphisms (SNPs) and the efficacy of antipsychotics (Pouget et al., 2014). Genetic studies have focused on the association between TRS and multiple candidate genes, involving multiple neurotransmitter systems that are consistent with the etiological hypothesis of TRS. It can be demonstrated by the pharmacodynamics of new antipsychotics affecting not only dopaminergic receptors but also serotonergic and glutamate receptors (Ji et al., 2008b; Kohlrausch et al., 2008; Bilic et al., 2014; Zink et al., 2014).

#### 3.1.1 Dopaminergic system

While altered dopaminergic function is the main feature of schizophrenia, patients with TRS might have distinct patterns in dopaminergic abnormality (Kogure et al., 2021). Specifically, as compared with patients with treatment-responsive schizophrenia, decreased dopamine synthetic capacity in striatum, decreased dopaminergic synaptic density and dopamine transporter protein expression in caudate nucleus have been reported in TRS patients (Demjaha et al., 2012; Sagud et al., 2018). It is well acknowledged that dopamine hypersensitivity induced by the compensatory upregulation of dopamine D2 receptors (DRD2) caused by the over-blockade of antipsychotics perhaps play a role in the pathogenesis of TRS (Potkin et al., 2020). Available evidence indicates that dopamine dysregulation associated with TRS may result from expression and/or functional changes in dopamine synthase, receptor, transporter, and catabolic enzyme (Purves-Tyson et al., 2017).

##### 3.1.1.1 COMT

Several studies have suggested that the COMT gene is known to be one of the key factors in the regulation of dopamine level in the PFC, which is associated with drug response. Besides, COMT is believed to be the carriers of the Met allele closely related to treatment resistance (Kirenskaya et al., 2018; Hajj et al., 2019). Met/Met of COMT was proved as predictors of TRS (Escamilla et al., 2018). It has been demonstrated that the occurrence of TRS tended to be higher in patients with the COMT L/L genotype than in the other patients. These findings suggested that the COMT genotype with low-activity is more common in TRS (Inada et al., 2003). Another study has identified the associations between COMT rs4680 and rs4818 polymorphisms and TRS, which were gender dependent. In female TRS patients, the COMT rs4680 AA genotype carriers were more frequent than G allele carriers, as the same with COMT rs4818 CC genotype. However, there was no such association in males (Sagud et al., 2018).

##### 3.1.1.2 DRD2, DRD3 and DRD1

The DRD2 density has been found to be lower in A1 allele carriers of Taq1A and Del allele noncarriers of—141C Ins/Del for DRD2 gene polymorphisms. Evidence suggested that haplotype polymorphisms of Taq1A and -141c Ins/Del DRD2 genes contributed to DRD2 antagonist resistance, especially linked to anxiety and depressive symptoms in schizophrenia (Kondo et al., 2003). For A-241G of DRD2, the G allele frequency was higher in the TRS patients than in the responsive schizophrenic group (Escamilla et al., 2018). Among the haplotypes observed in the DRD3 gene, the T/A/G/A/C haplotype was related to TRS (Kohlrausch et al., 2008). Besides, the Ser/Gly genotype of DRD3 may predict TRS (Escamilla et al., 2018). Data also showed that the sensitivity to treatment for schizophrenia was affected by DRD1 rs4532 polymorphism with the presence of G-allele increasing the risk of TRS (Ota et al., 2012).

#### 3.1.2 Serotonergic system

The pathogenesis of schizophrenia is related to the abnormality of the serotonergic system (Herken et al., 2003). Components of the serotonin system are being investigated as a risk factor for schizophrenia and are believed to play an important role in the clinical efficacy of antipsychotics (Lieberman et al., 1998). This is supported by the fact that some atypical antipsychotic drugs such as risperidone and clozapine modulate serotonin for antipsychotic effects (Roth et al., 2004). Altered serotonin responses in the brains of TRS patients have been reported (Anttila et al., 2007). Serotonin (5-HT) receptor and serotonin transporter (5-HTT) are the key components of the serotonergic neurotransmitter system (Wang et al., 2007). In recent years, the role of their gene polymorphisms in TRS has been studied. For example, clozapine exerts antipsychotic effects by binding to several serotonin receptor subtypes that can reduce dopaminergic neurotransmission (Ji et al., 2008b; Kohlrausch et al., 2010). It is easy to think of studying the relationship between serotonin receptor subtypes and TRS. However, in a study analyzed the mutations in three serotonin receptors (HTR2A, HTR3A, and HTR4), even though patients with the T/T genotype of the HTR3A polymorphism received significantly higher daily antipsychotic doses, no significant association was observed between TRS and each allele, genotype, and haplotype (Ji et al., 2008b).

##### 3.1.2.1 HTR2A and HTR3B

The 5-hydroxytryptamine receptor 2A (HTR2A) gene polymorphism was implicated to play a role in the pathogenesis of schizophrenia. Although the findings were mixed, HTR2A polymorphisms could predict TRS in general. Consistent with the previous result, evidence showed that T102C polymorphism in the HTR2A gene did not accelerate susceptibility to schizophrenia. However, the frequency of hospitalization was higher in the patients with T/C and T/T genotypes compared with the patients with C/C genotype, suggesting that it may represent a poorer prognosis and help distinguish TRS (Herken et al., 2003). Another study hypothesized that HTR2A T102C polymorphism, tryptophan hydroxylase 1 (TPH1) gene polymorphism and the G-protein beta-3 subunit (GNB3) C825T polymorphism alter serotonergic neurotransmission and are related to treatment response in schizophrenia. The HTR2A T102C C/C genotype or the TPH1 C/A genotype, and the GNB3 C825T C/T genotype were found in TRS patients when compared with responders. In addition, 5-HT2A T102C polymorphism and the GNB3 C825T C/T polymorphism were associated with genders (Anttila et al., 2007). Besides, the deletion allele at the 3 bp-insertion/deletion polymorphism site (-100_-102delAAG) encoding HTR3B was significantly more frequent in the TRS group than the insertion allele by a single-marker comparison (Ji et al., 2008a).

##### 3.1.2.2 5-HTT

The 5-HTT, encoded by the SLC6A4 gene, is responsible for reabsorption of serotonin into the presynaptic neuron and is a major regulator of serotonin function (Lesch et al., 1996). In addition, 5-HTTLPR is a 44 bp insertion/deletion polymorphism in the promoter region of SLC6A4, in which the short and long HTTRLP variants differentially modulate transcriptional activity of the 5HTT gene promoter (Sookoian et al., 2008). Another 5-HTT polymorphism is a variable number of tandem repeat elements known as HTTVNTR in the intron2. Compared with all other serotonin transporter-promoter region (SERT-PR) genotypes, “SS” SERT-PR carriers were less likely to develop TRS, while “LaS” SERT-PR carriers were almost three times more likely to develop TRS than those with the SS genotype (short‘S’ and long“L” allele) (Bilic et al., 2014). However, some inconsistent results were obtained. It was revealed that polymorphisms of rs6295 which encoded 5-hydroxytryptamine 1A receptor (5-HT1A) and 5-HTTLPR can influence some clinical symptoms of schizophrenia but no association was found in the progression of TRS (Terzić et al., 2015). Following risperidone treatment, the 5-HTTRLP patients without the L allele showed worse improvement on the Brief Psychiatric Rating Scale (BPRS) than patients with the L allele but no such relationship was found for the HTTVNTR. In haplotype analysis, the frequency of L-12 (the size of HTTVNTR fragment was 300bp) haplotype showed significant differences between responders and non-responders (Wang et al., 2007).

##### 3.1.2.3 Combined genetic effects of serotonin and dopamine

The combined role of genes in the serotonin and dopamine systems has been reported in several studies (Kohlrausch et al., 2008; Zink et al., 2014). TRS is attributed to an interaction between the dopamine transporter (DAT) and a variable number tandem repeat (VNTR) polymorphism inside intron 2 (SERT-in2). Patients with SERT-in2ll and DAT 9/10, 9/9, 9/9, and 6/6 genotypes were more likely to develop TRS, as were those with DAT 10/10 or 10/12 genotypes paired with SERT-in2ls or ss genotypes (Bilic et al., 2014).

#### 3.1.3 Glutamatergic and GABAergic system

The dopaminergic system of schizophrenia has been studied for a long time, which can indeed explain some pathogenesis and therapeutic targets of schizophrenia. However, it is accepted that dopamine dysfunction cannot explain all aspects of schizophrenia (Bartolomeis et al., 2019). A large number of studies have shown that the pathophysiology of schizophrenia involves not just dysregulation of dopaminergic, but aberrant γ-aminobutyric acid (GABA), glutamatergic neurotransmission and their interactions (Lin et al., 2012). Several studies of pharmacological models and clinical trials have elucidated the role of excitatory glutamatergic signaling in the pathophysiology of schizophrenia from the angles of neurodevelopment risks, pathologic precipitations, and genetic susceptibility (Lin et al., 2012; Zink et al., 2014; Uno and Coyle, 2019). There is considerable evidence indicating that treatment resistance is associated with elevated glutamate levels without altered dopaminergic function (Demjaha et al., 2014; Zink et al., 2014). In support, a series of cross-sectional studies have shown that poorer antipsychotic responses are associated with higher levels of glutamate metabolites in the anterior cingulate cortex (ACC) compared to patients who exhibit good responses or healthy volunteers (Leung et al., 2019; Tarumi et al., 2020; Egerton et al., 2021). Higher levels of glutamate metabolites also predicted the adverse effects after re-initiation of antipsychotics (Egerton et al., 2021). Inhibitory GABAergic system has also been reported to participate in TRS (Tan et al., 2019; Bojesen et al., 2020). The GABAergic system dysfunction is associated with cognitive dysfunction and patients with TRS often have severe cognitive impairment, suggesting that GABAergic dysfunction may be more closely related to TRS patients (Miyazawa et al., 2022).

##### 3.1.3.1 NMDA

N-methyl-D-aspartic acid (NMDA) and α-amino-3-hydroxy-5-methyl-4-isoxazolepropionic acid (AMPA) receptors are the key components of glutamatergic neurotransmission (Uno and Coyle, 2019). Multiple evidence suggested that the N-methyl D-aspartate (NMDA) receptor mediated function is reduced in schizophrenia (Lin et al., 2012). For example, the NMDA receptor antagonists, PCP and ketamine, induce psychiatric and behavioral changes related to schizophrenia, even TRS in animals models (Moghaddam and Javitt, 2012; Rajagopal et al., 2022). An interesting study aimed to examine whether polymorphisms in the 366C/G and 2664C/T genes of glutamate ionotropic receptor NMDA type subunit 2B (GRIN2B) were associated with TRS in Chinese patients. Although no significant association was found, the results suggested that patients with GRIN2B 2664C/C genotype require a higher dose of clozapine (Chiu et al., 2003).

##### 3.1.3.2 GRM3

There is also some indication that the glutamate metabolism receptor 3 (GRM3) gene, which controls the signaling of NMDA receptors, is relevant to antipsychotic response. Two GRM3 markers (rs1989796 and rs1476455) were linked to the refractory global symptoms. Specifically, participants with an rs1476455_CC genotype or rs1989796_CC genotype had significantly higher BPRS scores than A-carriers or T-carriers respectively, as indicated in a cohort of ninety-five TRS patients (Bishop et al., 2011). Another study revealed that GRM3 rs1468412 was linked to the changes in cognition and symptom response after antipsychotic medication, potentially assisting in the identification of patients with poor symptom improvement (Bishop et al., 2015). Furthermore, data suggests that synergistic association of PI4KA and GRM3 genetic polymorphisms may influence antipsychotic reactions (Kaur et al., 2014).

##### 3.1.3.3 GAD1 and GABBR2

An association study for five SNPs including Glutamate decarboxylase 1 (GAD1), Gamma-Aminobutyric Acid Type B Receptor Subunit 1 (GABBR1) and Gamma-Aminobutyric Acid Type B Receptor Subunit 2 (GABBR2) genes was performed among 357 patients with TRS, 682 non-TRS patients and 508 healthy controls. The findings demonstrated that rs3749034 on GAD1 and rs10985765 on GABBR2 could be related to TRS. However, no significant differences in allelic and/or genetic distributions for any of the five SNPs were revealed. Thus, further studies in other ethnic groups and with larger samples are needed (Miyazawa et al., 2022). Interestingly, the evidence confirmed that the interaction between dopaminergic signaling and GABAergic gene expression affects the clinical phenotype of schizophrenia which can help identify TRS patients. Specifically, the percentage of subjects with Met allele of rs4680 on the COMT gene and C/C homozygote of rs3470934 on the GAD1 gene was significantly higher in the TRS group than non-TRS group and HC group (Kogure et al., 2021).

#### 3.1.4 Other gene variants

##### 3.1.4.1 Muscarine

Muscarinic activity might contribute to the clinical signs and symptoms of schizophrenia. Type I muscarinic receptor (CHRM1) is the prefrontal cortex’s main cholinoceptive target and linked to cognitive performance (Marino et al., 1998). C267A is one of the genes that codes for it. The heterozygote group (267C/A) performed better than the homozygote group (267C/C) in terms of accurate replies and persistent errors of the Wisconsin card sorting test. However, the genotype distribution of schizophrenia patients in this study did not differ from that of healthy controls, proving that the CHRM1 C267A polymorphism did not significantly increase the susceptibility to schizophrenia (Liao et al., 2003).

##### 3.1.4.2 Cannabinoid

Neurobiological studies have shown that the endocannabinoid system plays a significant role in both the propensity for schizophrenia and the efficacy of the response to antipsychotic medicines (Fernandez-Espejo et al., 2009). Endogenous cannabinoids bind to and activate two distinct receptors: the mostly central cannabinoid receptor type 1 (CB1) and the primarily peripherally expressed cannabinoid receptor type 2 (CB2) (Ishiguro et al., 2010). The majority of studies concentrated on the CB1 gene polymorphisms (Ferretjans et al., 2012). Only one investigation supported the association between cannabinoid receptors and drug responsiveness. The 1359 G/A (rs1049353) G allele of the CB1 gene was much more frequent in non-responsive individuals than in responsive patients (Hamdani et al., 2008). There was no correlation between the reaction to antipsychotics and three additional genetic variants (rs806368, rs806379, and rs806380) coding the CB1 (Hamdani et al., 2008).

##### 3.1.4.3 Histamine

The H1, H2, H3, and H4 histamine receptors are activated by the central nervous system (CNS) histaminergic system, which controls sleep-wake, attention, and metabolic balance (Raddatz et al., 2012). The evidence found the efficacy of risperidone was affected by the histamine receptor H4(HRH4) polymorphism rs4483927. Patients with the TT genotype of rs4483927 had poor Positive and Negative Syndrome Scale (PANSS) scores. However, because 113 Chinese Han patients with schizophrenia who had never before encountered antipsychotic drug resistance followed an 8-week course of risperidone monotherapy in this study, no concrete proof of histamine receptor variation for TRS was offered (Wei et al., 2013).

##### 3.1.4.4 Cell signaling

The cell signaling and neural development is modulated by AKT1 gene which is a member of the serine-threonine protein kinase. This disorder is associated with developmental, structural, and functional abnormalities of the hippocampus (Balu et al., 2012). AKT1 genes were upregulated in the TRS group in comparison to the healthy group. However, the other genes including neurotransmission, inflammation, neurodevelopment and protein degradation were not found positive results (Moretti et al., 2018).

#### 3.1.5 Future perspectives in genetic biomarkers of TRS

The dopaminergic, serotonergic, and glutamate/GABA systems have been the focus of recent advances in pharmacogenetics to identify specific risk genes for TRS. There is only weak evidence that genetic markers for other systems such muscarinic, histamine, cannabinoids, etc. are involved in TRS. However, certain findings supported the discovery of genes associated with risk for schizophrenia and the pathophysiology of schizophrenia (Scarr et al., 2013; Brown et al., 2014). These regions now serve as a starting point for investigation into genetic TRS markers and pharmaceutical targets.

The genetic risks of TRS and the underlying mechanisms are more likely to involve the superposition or interaction of multiple genetic loci (Ruderfer et al., 2016; Samanaite et al., 2018). Genome-wide association studies (GWAS) becomes an important tool for understanding the biological underpinnings of schizophrenia. The combinations of the risk functional SNPs observed in this study may be useful as new biomarkers in clinical settings. Pharmacogenomic studies use genome-wide data instead of a candidate gene approach to understand the mechanisms of antipsychotic response and are being applied to identify polymorphisms in TRS (Ackenheil and Weber, 2004; McClay et al., 2011). Polygenic risk score (PRS)analysis captures the genetic load of alleles associated with traits across multiple loci representing an approximation of the genetic risk burden (Euesden et al., 2015), and thus can aggregate the effects of multiple SNPs on the genome to create the sum of alleles associated phenotype through GWAS (Nucifora et al., 2019). There is some evidence that a greater genetic burden equates to a greater likelihood of developing TRS (Nucifora et al., 2019; Pisanu and Squassina, 2019). However, most studies only used PRS method to investigate risk variants in schizophrenia, and no GWAS studies specifically conducted for TRS. It is speculated that the development of a PRS specifically for TRS may show higher predictive accuracy.

In spite of SNPs, rare mutations are also shown to increase the risk of schizophrenia (O'Tuathaigh et al., 2017; Purcell et al., 2014). Genes encoding calcium channels, and proteins involved in glutamatergic neurotransmission and synaptic plasticity have been independently implicated (Fromer et al., 2014; Purcell et al., 2014). The role of rare disruptive mutations was extensively investigated in TRS. Compared with the non-clozapine treated patients (treatment responsive), the 347 gene targets of antipsychotic drugs were enriched for singleton disruptive mutations in TRS patients (Cao et al., 2020). Another study reported a large exome sequencing study of *de novo* mutations in schizophrenia, comprising 482 nonsynonymous mutations, in which 64 were *de novo* loss-of-function (LoF) occurred preferentially in schizophrenia cases with premorbid cognitive impairment (Fromer et al., 2014). Interestingly, they also found overlap between genes affected by rare variants in schizophrenia and those within GWAS loci, suggesting that common and rare variants are complementary rather than contradictory (Schizophrenia Working Group of the Psychiatric Genomics Consortium, 2014).

Copy number variants (CNVs) are essentially deletions and duplications in the human genome that fall between large (chromosomal aberrations >1 Mbp) and small (insertions or deletions 1–50 bp) aberrations (Girirajan et al., 2011). Recent studies of schizophrenia (SCZ) have reported an increased burden of *de novo* CNVs (International Schizophrenia Consortium, 2008). The relationship between CNV and TRS was supported by two studies. A high-resolution genome-wide CNV analysis was carried out on a predominantly Japanese population with 1699 schizophrenia patients and 824 controls using array comparative genomic hybridization. TRS was seen in individuals with a 22q11.21 deletion XXX/XXY, and two clinically significant CNVs (Kushima et al., 2017). This result supported patients with clinically significant CNVs were more likely to experience TRS. Another study confirmed a connection between the overall amounts of genome-wide duplications and TRS in 612 schizophrenia patients. The results suggested an increase in rare replication burden across the genome of TRS patients (Martin and Mowry, 2016).

Although the two studies did not include TRS as a separate subgroup, they provided detailed evidence for CNV involvement in the pathogenesis of schizophrenia. The first study measured the amount of fragment of the human satellite III (f-SatIII) in leukocytes from 401 healthy individuals and 840 schizophrenia patients (Ershova et al., 2019). The patients generally differ from the healthy group significantly because their repeat content values were lower. Another study reported that in contrast to 3181 controls, 4 out of 3391 schizophrenia patients had the serine/threonine kinase gene unc-51-like kinase 4 (ULK4) deletions spanning exons 21 to 34. The big splice variant of ULK4’s deletions that remove exons 33 and 34 were also more prevalent in schizophrenia patients (Lang et al., 2014).

We also found some evidence that involves epigenetics including DNA methylation and microRNAs (miRNAs) (You et al., 2020; Hannon et al., 2021). One concrete example was the ribonuclease DICER1, which plays a key role in the production of miRNAs. In comparison to the healthy group, DICER1 genes were increased in the TRS group (Moretti et al., 2018). The top three miRNAs with the highest fold change values were screened out from peripheral blood mononuclear cells, hsa-mir-218-5p and hsa-mir-1262 of them were biomarkers for an earlier and more accurate diagnosis of TRS (You et al., 2020). It is interesting to note that, compared to non-TRS instances, TRS cases appear to have much greater proportions of granulocytes and lower proportions of CD8^+^ T-cells based on cellular composition variables derived from DNA methylation data. TRS was linked to considerably higher smoking score determined from DNA methylation than non-TRS (Hannon et al., 2021).

To sum up, the development of a PRS specifically for TRS through GWAS, the mechanism-driven approach to identify rare mutations and the epigenetics will provide more information for TRS in addition to exploring candidate genes SNPs (Schizophrenia Working Group of the Psychiatric Genomics Consortium, 2014; Pisanu and Squassina, 2019; Pardiñas et al., 2022). Much of the evidence for the types of these genetic variants focuses on schizophrenia now, and exploring the association of TRS with these variants as a separate subgroup would provide a more thorough genetic model for TRS.

### 3.2 Inflammation

Immune reactions and inflammation have long been implicated in the pathophysiology of schizophrenia, according to the available epidemiological, clinical, and postmortem evidence (Bechter et al., 2010). Prenatal and early life are vulnerable periods for precipitating factors, like infection, autoimmune or psychological stress to result in inflammation at later stage of mental illness (Möller et al., 2015). For example, abuse in childhood may have long-term consequences, leading to psychotic onset during adolescence or adults (Leboyer et al., 2016). Systemic immune-inflammatory cells can pass through the blood-brain barrier, altering central inflammatory processes, particularly in astrocytes and microglia (Leonard and Maes, 2012). Moreover, increased peripheral and cerebral inflammatory processes were found to play a role in TRS (Roomruangwong et al., 2020). Inflammatory indicators are linked to cognitive deficits and negative symptoms in schizophrenia, both of which are notoriously difficult to treat (Garcia-Rizo et al., 2012). Previous research has similarly associated higher inflammatory status with a worse clinical outcome in TRS patients (Momtazmanesh et al., 2019). More than 60% of TRS patients had monocyte activation and proteome changes in blood, as well as moderate cerebrospinal fluid pathology, microglia activation, or dysconnectivity in neuroimaging (Bechter, 2013).

#### 3.2.1 Complete blood count

Biomarkers of psychotic condition can be traced in peripheral immune system with different phenotypes stratified by blood cells (Miller et al., 2013). There are few studies focusing on the relationship between therapeutic responsiveness and inflammatory complete blood count (CBC) markers in patients with schizophrenia. Schizophrenia patients treated with clozapine had increased relative numbers of NK cells, naive B cells, CXCR5^+^ memory T cells and classical monocytes, while had decreased numbers of dendritic cells (DC), HLA-DR^+^ regulatory T-cells (Tregs), and CD4^+^memory T cells. More severe negative and cognitive symptoms were linked to the above mentioned decreased cells (Fernandez-Egea et al., 2016). In clinical settings, the neutrophil to lymphocyte ratio (NLR), monocyte to lymphocyte ratio (MLR), and platelet to lymphocyte ratio (PLR) derived from CBC data can be used as proxies for systemic inflammation. In a six-year retrospective study, although baseline NLR was similar, only the responsive subgroup showed decrement in NLR after treatment, suggesting that persistent inflammation is associated with treatment resistance in schizophrenia. Meanwhile, PLR was higher on admission in the responsive group and significantly decreased after treatment, while no change was observed in the TRS patients due to lower PRL at admission (Labonté et al., 2022). These findings suggested that CBC could be a feasible biomarker and used to track TRS patients for targeted therapies (Fernandez-Egea et al., 2016; Labonté et al., 2022).

#### 3.2.2 Cytokines

The immune response is largely regulated by interleukin-6 (IL-6), which plays a vital role in the immunological response during the body’s anti-infection process. A great number of studies on this cytokine have consistently found that TRS patients have considerably greater serum IL-6 levels than normal controls or treatment-responsive patients (Maes, 2000; Roomruangwong et al., 2020). Interestingly, elevated IL-6 levels were found to be inversely related to the endogenous anti-cytokine CC16, which may constitute a hallmark of schizophrenia (Lin et al., 1998). Dickkopf-related protein (DDK1) and IL-6 levels in the non-responders to treatment group were significantly higher than in the partial responsive and control groups. Further meta-logistic regression analysis revealed that IL-6 in combination with CCL11 (eotaxin) was the greatest predictor of non-responders when compared to partial-responders (Al-Dujaili et al., 2021). In addition, IL-6 may result in downregulation of Wnt signaling, thus leading to a disruption of the blood-brain barrier (BBB) and neuroinflammation (Al-Dujaili et al., 2021). TRS appeared to be linked to other elevated cytokine levels of IL-12/IL-23p40, IL-17A, and beta 2 macroglobulin (B2M) in blood. These cytokines have a role in the establishment and differentiation of Th17 immune pathways, suggesting that the activation of the IL-17 pathway may present at onset of the disease and increase as the disease progresses to drug resistance (Leboyer et al., 2021). Besides, several other studies patients with TRS had higher levels of IL-2, IL-8, IL-10, soluble tumor necrosis factor receptor 1 (sTNF-R1), sTNF-R2, monocyte chemotactic protein 1 (MCP-1) and IL-1R antagonist (IL-1RA) than the control group, indicating immunological inflammation is one of the important components of TRS (Lin et al., 1998; Maes, 2000; Zhang et al., 2004; Miller et al., 2013; Noto et al., 2015; Al-Dujaili et al., 2021; Leboyer et al., 2021; Labonté et al., 2022).

#### 3.2.3 CRP and telomere attrition

Furthermore, evidence shows that individuals with high levels of C-reactive protein (CRP), a biomarker of chronic or acute inflammation, were more likely to develop schizophrenia (Horsdal et al., 2017). When compared to healthy controls, TRS patients had considerably higher levels of high-sensitivity C-reactive protein (hsCRP)(Challa et al., 2021). However, they discovered no link between CRP levels and TRS at the time of the first schizophrenia diagnosis (Horsdal et al., 2017). Finally, patients with severe mental illnesses had shorter telomeres and more inflammation. It revealed that telomere attrition was also caused by a number of biological injuries, including inflammatory processes (Squassina et al., 2020).

In short, a growing body of evidence supported the association of inflammatory markers with disease risk, severity, and treatment resistance of schizophrenia. Finally, due to the role of the immune system in the physiopathology of schizophrenia and the relative availability of testing methods, immune markers are considered as potential peripheral biomarkers for TRS patients.

### 3.3 Stress

#### 3.3.1 Oxidative stress

Oxidative stress has been repeatedly identified as one of the most important biological underpinnings of the pathophysiology of schizophrenia (Cui et al., 2020), which is evident by malfunction of antioxidant system and the change of activity of different antioxidant enzymes (Pinheiro et al., 2017). The levels of antioxidant, catalase, and glutathione peroxide are reduced in schizophrenia. It has been found that antioxidative status is negatively associated with specific cognitive abnormalities, whereas glutathione levels are positively correlated with executive function (Martínez-Cengotitabengoa et al., 2012). Because of its abundance of polyunsaturated fatty acids, high consumption of oxygen, and low quantities of antioxidative enzymes, the brain is particularly susceptible to oxidative stress (Möller et al., 2015). Reduced antioxidant levels in the brain, combined with increased production of reactive oxygen species, put neurons in greater danger of injury, especially in TRS (Pinheiro et al., 2017).

##### 3.3.1.1 Lipid peroxidation

In TRS patients, lipid peroxidation and neuronal damage were shown to be higher than in non-refractory individuals (Medina-Hernández et al., 2007). This study found elevated serum levels of lipid peroxidation byproducts malondialdehyde (MDA) and 4-hydroxynonenal (4-HNE) and neuron-specific enzymes in TRS patients compared with healthy controls indicated greater systemic oxidative stress and greater neuronal metabolic changes in TRS patients (Keller et al., 1999; Medina-Hernández et al., 2007). Moreover, telomere length shortening in peripheral leukocytes is a marker of poor treatment response in patients with chronic schizophrenia, which may be caused by oxidative stress-induced cellular dysfunction leading to the exacerbation of TRS (Yu et al., 2008).

##### 3.3.1.2 Membrane phospholipids

From another perspective, the composition of fatty acids is associated with neurocognition and mood regulation in the brain (Assies et al., 2014). Changes in enzyme-induced and non-enzyme-induced antioxidant systems generated increased lipid peroxidation, leading to reduced levels of fatty acids in schizophrenia (Bitanihirwe and Woo, 2011). For example, oxidative stress reduced the content of polyunsaturated fatty acids (especially omega-3 polyunsaturated fatty acids) and increased lipid peroxidation byproducts (Assies et al., 2014). Messamore and McNamara collected more than 130 blood samples from psychosis patients with treatment-resistant mood disorders. The majority of these patients had extremely low levels of eicosapenaenoic acid (EPA) and docosahexaenoic acid (DHA), indicating long-chain omega-3 fatty acid deficiency in these patients (Messamore and McNamara, 2016). Our group studied the contents of fatty acids in the erythrocyte membrane of schizophrenia. Patients were grouped into different episodes, including the first episode, 2–3 episodes, 4–6 episodes, and more than 6 episodes. Overall, there were reductions in both saturated and unsaturated fatty acids at baseline compared with healthy controls. Follow-up assessment after 1 month showed an increase in membrane fatty acids in patients treated with atypical antipsychotics only within 3 episodes. In addition, changes in C22:5n3 and omega 3 index, as well as gender and frequency of episodes, were all significant risk variables for diminished treatment response. Using a targeted metabolomics approach, the study revealed abnormalities in fatty acid metabolism that lead to a reduced response to treatment in patients with multiple schizophrenia (Li et al., 2022).

#### 3.3.2 Carbonyl stress

Carbonyl stress is a metabolic abnormality characterized by increased reactive carbonyl compounds (RCOs) and their attendant protein modifications, which leads to the accumulation of advanced glycation end products (AGEs) such pentosidine (Ohnuma et al., 2018). Oxidative stress is a central mediator of AGEs formation which converts glucose and lipids to reactive carbonyl compounds (RCOs), and RCOs are transformed to AGEs and advanced lipid peroxidation end products (Ohnuma et al., 2018). Glyoxalase breaks down the RCO into lactic acid and glutathione for detoxification and the rate-limiting enzymes in this metabolic pathway are glyoxalase 1 and 2 (GLO1 and GLO2)(Kouidrat et al., 2015; Rodrigues et al., 2020). Besides, Pyridoxamine, one of the three forms of vitamin B6, can detoxify RCO, including AGEs (Li et al., 2022). There is increasing evidence that carbonyl stress is closely associated with schizophrenia accompanied by exhibiting high plasma pentosidine and low serum vitamin B6 levels (Miyashita et al., 2014).

In the first study to observe the clinical features of schizophrenia with increased carbonyl stress, patients with carbonyl stress were found to be less educated, have significantly higher rates of hospitalization, stay longer in hospital, and take higher doses of antipsychotic medications than patients without carbonyl stress (Miyashita et al., 2014). The severe clinical symptoms identified in these carbonyl stress patients are very comparable to the definition of TRS (Kane et al., 1988). Serum pyridoxal, which is measurably stable, is converted from pyridoxamine (Son et al., 2020). Another study from the same research group also mentioned that high pentosidine and low pyridoxal levels in the peripheral blood could be state markers of TRS (Katsuta et al., 2014). Pyridoxal levels have been shown to decline with clinical progression and are linked to less symptom alleviation, whereas pentosidine levels are higher, possibly due to higher antipsychotic doses (Katsuta et al., 2014).

#### 3.3.3 Limitations of stress-related biomarkers for TRS

Metabolites, intermediates, enzymes and their activity in many metabolic pathways are altered due to high levels of reactive oxygen species (ROS)(Nedic Erjavec et al., 2018). However, there are only a few studies on the role of this process in the TRS. As summarized above, enzymatic (superoxide dismutase (SOD) and catalase) and non-enzymatic antioxidants (e.g., glutathione (GSH)) may participate in this pathological process (Do et al., 2009). The relationship among neuronal and systemic oxidative stress, lipid peroxidation and treatment-resistant psychiatric disorders is largely unknown. Hopefully, the focus on carbonyl stress in the field of schizophrenia has broadened the scope of therapeutic resistance markers and neurobiology for schizophrenia.

Besides, some evidence suggests that therapeutic approaches for alleviating carbonyl stress, such as vitamin B6 supplementation, could offer novel therapeutic benefits (Miyashita et al., 2014). In a 24-week open trial, ten Japanese schizophrenic patients with high plasma pentosidine were recruited and given a high dose of pyridoxamine. According to the findings, this medication is a viable treatment for TRS caused by elevated carbonyl stress (Itokawa et al., 2018). Several other clinical studies have also looked into the effectiveness of vitamin B6 supplementation in schizophrenia, however the results were mixed (Miyashita et al., 2014). In the future, prospective and longitudinal designs are needed for carbonyl stress to clarify the exact clinical relationship between TRS and two proposed biomarkers, as well as to validate these new findings, such as pyridoxamine supplementation, which may have new therapeutic benefits for this subset of patients.

## 4 Peripheral biomarkers of TRS with clozapine treatment

### 4.1 Genetic biomarkers

It is likely that clozapine impacts a complex network of neurotransmitter pathways given its affinity for various targets (Miller, 2009). Clozapine is known to exert its action on adrenergic, histamine, and muscarinic receptors in addition to the three primary neurotransmitter systems indicated above (Nair et al., 2020).

#### 4.1.1 Dopaminergic system

Rudi Hwang et al. concentrated on the reaction to clozapine and the dopamine receptor gene polymorphisms. Their findings were unique in that they were based on diverse populations consisting of 232 subjects with 183 Caucasians and 49 African Americans. Three dopamine receptors tested in the African American population showed promising findings. The A/C genotype of SNP rs265976 in DRD1 was more prevalent in non-responders (Hwang et al., 2007). The impact of 12 SNPs across the whole DRD2 gene on clozapine response was examined in this exploratory investigation. Being a responder was linked to having the allele 2 (B2) of TaqIB, allele A of rs1125394, and not having the TaqIA1 (T) allele (Hwang et al., 2005). Additionally, it was found that the DRD4 120-bp 1-copy allele and intron I (G)n 142-bp/140-bp genotype were related to non-responder status (Hwang et al., 2012). Two dopamine receptor gene polymorphisms were found in Caucasian individuals. The DRD3 rs2134655 A allele was linked to improved responsiveness. The T allele of rs1394016 and the GG genotype of rs2399504 both showed the same tendency (Hwang et al., 2010). Besides, exon III 4R allele carriers responded better than non-4R allele carriers. All DRD5 tests were negative (Hwang et al., 2012).

There was additional evidence that the reaction to clozapine is related to dopamine receptors. The 2,2 DRD1 homozygotes have responded to clozapine with a more BPRS symptom improvement compared to 1,2 DRD2 subjects (Potkin et al., 2003). A recent genome-wide association study found an SNP, rs2514218, 47 kb upstream of the DRD2 gene, was linked to risk for schizophrenia. The rs2514218 risk allele A was linked to a larger decline in BPRS scores in the Caucasian group with clozapine (Huang et al., 2016). Compared to the D2 receptor, the DRD3 receptor exhibits a higher affinity for the clozapine. In comparison to the responders, the non-responders had a much greater genotype of DRD3 rs9680 AA gene (M et al., 2020). A Ser9Gly point mutation that changes the amino acid serine (Ser-9) to glycine (Gly-9) in the N-terminal extracellular region of DRD3. Ser-9/Ser-9 genotypes were prevalent in non-responders, and Ser-9/Gly-9 genotypes were prevalent in respondents (Scharfetter et al., 1999).

Two studies discovered a link between the COMT Val158Met polymorphism and clozapine responses. In the first study, clinical responses to clozapine were superior in those Val/Val patients compared to the val/met or met/met patients who had one or two met alleles in the PANSS Negative Subscale (Bosia et al., 2015). The second investigation did not discover an independent relationship between the COMT Val158Met polymorphism and clozapine responsiveness. However, participants with the COMT Val/Met or Met/Met genotype as well as the DRD4 120-bp alleles (120/240 and 120/120) demonstrated a significantly improved clinical response to clozapine (Rajagopal et al., 2018).

#### 4.1.2 Serotonergic system

The serotonergic system appears to be the primary mechanism through which clozapine exerts its effects, and changes in serotonergic synaptic levels may have an impact on the antipsychotic response (Kohlrausch et al., 2010). The link between the serotonin and dopamine systems is mediated by the 5-HT1A-R -1019 C/G polymorphism. There was a higher improvement in the PANSS Negative Subscale in 5-HT1A-R G/G patients with clozapine treatment (Bosia et al., 2015). The association between 5-HT2A polymorphisms and clozapine responsiveness was verified by three trials, and the outcomes were consistent (Arranz et al., 1996; Arranz et al., 1998; Masellis, 1998). The frequency of the allele Tyr452 described in the coding region of the 5-HT2A receptor gene and homozygosity for the allele G-1438 identified in the promoter region were both higher in non-responders than in responders (Arranz et al., 1998). The 5-HT3A receptor, which is the only receptor-coupled ion channel among the serotonin receptors, has emerged as a key target in schizophrenia patients (Souza et al., 2010; Rajkumar et al., 2012). It is well documented that clozapine treatment responsiveness has been linked to alleles of three HTR3A variations, including rs2276302, rs1062613, and rs1150226. The G-T-T haplotype, which spans rs2276302-rs1062613-rs1150226, was correlated to a poorer response (Souza et al., 2010). Consistent with this, this study also discovered that minor alleles of both rs1062613 and rs2276302 were closely linked with clozapine responses. What is innovative about this study is that HTR3A pharmacogenetics combined with clinical predictors can more strongly explain differences in clinical response to clozapine (Rajkumar et al., 2012). Additionally, because HTTLPR regulates the transcriptional activity of the 5-HTT gene, genotypes with low expression such S'/S′ or S'/L′ would be more likely to have poor responses to clozapine (Kohlrausch et al., 2010).

#### 4.1.3 Glutamate/GABA system

Clozapine affects multiple glutamate/GABA signal transduction receptors, transporters, and enzymes, drawing attention to the interaction between SNP gene variations and clozapine sensitivity in these pathways (Daskalakis and George, 2009; Gammon et al., 2021). However, there are no ideal positive results on the relationship between the variation of genes in this system and clozapine response. Using standard genotyping procedures, the relationship between clozapine response and ten glutamate system gene variants of GRM2, SLC1A2, SLC6A9, and GAD1 (encoding metabotropic glutamate receptor 2 (mGluR2), glutamate transporter 1 (GLT1), glycine transporter 1 (GlyT1), and GAD1, respectively) was investigated. Surprisingly, no significant association was observed except that CC homozygous of rs16831558, which is located in the GlyT1 gene (SLC6A9), showed allele-dose-dependent improvement in positive symptoms as compared with T allele carriers (Taylor et al., 2017). In another study, whereas the data showed that an allele carrier of the GRIN2B intron variant rs1072388 responded to clozapine slightly better than G homozygous, there was no significant correlation between the seven GRIN2B variants and treatment response to clozapine (Taylor et al., 2016). Another study that included 45 patients taking clozapine was unable to confirm the presence of any of the four single-nucleotide variations in the GRIN1, GRIN2A, or GRIN2B subunits of the NMDA-R.

#### 4.1.4 CYP

It has been suggested that variations in cytochrome P450 (CYP) enzyme activity have a major impact on the pharmacokinetics of clozapine. Several cytochrome P450 (CYP) enzymes, including CYP1A2, CYP2C19, CYP2D6, and CYP3A4, have been shown to involve clozapine metabolism. The low CYP3A4 express patients needed a much lower dose (50%) of clozapine to achieve the ideal blood level than other expressers (Tóth et al., 2017). When compared to the control group and those who responded to clozapine, the distributions of alleles and genotypes revealed a higher frequency of CYP2C19*17 in the patients who had partial response to clozapine (Rodrigues-Silva et al., 2020). The genotypes of CYP1A2 and CYP2D6 expression in the patients were not found positive results (Tóth et al., 2017).

#### 4.1.5 FKBP5, NTRK2, ABCB1 and ITIH3

Because clozapine affects the Hypothalamic–pituitary–adrenal (HPA) axis, the FK506 binding protein 5 (FKBP5) modulating HPA axis is of particular importance. When compared to C-carriers, FKBP5 rs1360780 TT-homozygotes had a 2.11 times higher risk of nonresponse. Neurotrophic tyrosine kinase receptor 2 (NTRK2) is BDNF’s high-affinity receptor. When compared to C-carriers, TT-homozygous for rs1778929 in the NTRK2 gene showed a 1.7-fold increased risk of nonresponse. In contrast, rs10465180 CC-homozygous individuals had a 2.15-fold increased risk of nonresponse compared to T-carriers (Mitjans et al., 2015). Adenosine triphosphate-binding cassette class B (ABCB1) is necessary for the passage of clozapine through a number of bodily barriers. The non-responder subgroup had a considerably higher frequency of the TT genotype allele than the responder subgroup (M et al., 2020). A genome-wide investigation identified risk loci that have an impact on each of the five main psychiatric disorders. The intronic rs2535629 polymorphism in Inter-alpha-trypsin inhibitor heavy chain H3 (ITIH3) is the region’s strongest association discovery (Cross-Disorder Group of the Psychiatric Genomics Consortium, 2013). This variation has also been linked to schizophrenia in Japanese patients. Patients with homozygous for the minor A allele of rs2535629 had the highest improvement in their negative BPRS scores after receiving clozapine medication (Brandl et al., 2016).

### 4.2 Other biomarkers

Brain-derived neurotrophic factor (BDNF) is crucial for neurogenesis and maturation of neural developmental pathways (Yamamori et al., 2013). A study found that compared to non-responders, responders had higher serum levels of BDNF (Krivoy et al., 2018). The study discovered that the start of clozapine treatment in patients with TRS was highly associated with a brief increase in WBC, neutrophil, and platelet count. The study showed that clozapine had early hematological effects, but it was not determined if these changes predict how a patient will respond to treatment (Blackman et al., 2021). When compared to non-TRS patients, those who did not respond to clozapine had higher levels of IL-12/IL-23p40, IL-17A, IL-6, IL-10, IFN, and B2M (Leboyer et al., 2021). The relationship between lipid dysregulation and the therapeutic response to clozapine in TRS was investigated. A rise in triglyceride levels was highly indicative of both clinical and PANSS score improvements (Lally et al., 2013). It was found that lower CLZ: NDMC (N-desmethylclozapine, the main metabolite of clozapine) ratios were related to higher cognitive function. This result indicated the CLZ:NDMC ratio may be helpful for maximizing clozapine’s potential cognitive advantage (Costa-Dookhan et al., 2020).

As we searched for neurotransmitter data, we discovered that histamine and muscarinic receptor pathways had been implicated increasingly (Mancama et al., 2003). However, the majority of clozapine and these receptor pathways evidence was being conducted on animals examining the mechanism of clozapine in these systems (Digby et al., 2012). The acetylcholine (ACh) innervation of five different receptor subtypes, CHRM1-CHRM5, causes the muscarinic system’s actions (Vilaró et al., 1994). Muscarinic pathways are crucial for memory, learning, and attention (Hagan et al., 1987). NDMC has been demonstrated to be an M1 muscarinic receptor partial agonist while clozapine was an M1 antagonist *in vitro* and *in vivo*, it is probable that NDMC agonism leads to the drug’s particularly advantageous effects (Davies et al., 2005; Li et al., 2005). Through M1 receptor activation, NDMC promoted the release of ACh in brain areas important for cognition, including prefrontal cortex and hippocampus (Carruthers et al., 2015). NDMC ameliorated novel object recognition impairment induced by phencyclidine, and these findings provided more evidence that M1 agonism was a possible target for the treatment of cognitive impairment in schizophrenia (Miyauchi et al., 2017). Additionally, we discovered some evidence that, at clinically meaningful concentrations, clozapine and its active metabolite NDMC interact with the four human histamine receptors (Mancama et al., 2002; Humbert-Claude et al., 2012). The literature’s findings, like those for muscarine, mostly explored the mechanism of this receptor in animal studies. There are few clinical studies on peripheral markers of these two major receptor pathways to predict or reflect the therapeutic effect of TRS.

## 5 From peripheral biomarkers to neurobiology of TRS

**FIGURE 2:**
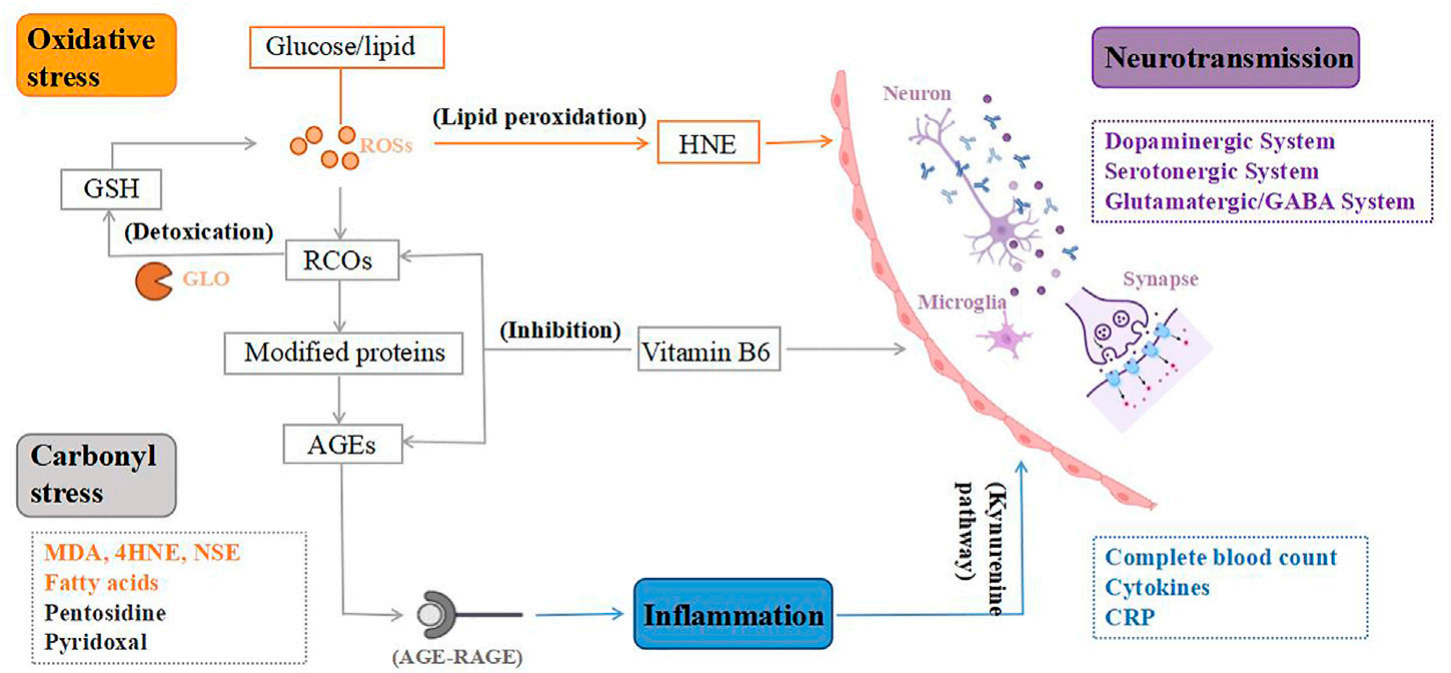
From peripheral biomarkers to neurobiology of treatment-resistant schizophrenia (TRS). To begin with, the lipid peroxidation product 4-hydroxynonenal (HNE) created by oxidative stress can alter the blood-brain barrier, and peroxidation also causes aberrant fatty acid levels in the synaptic membrane, leading to an altered neuronal microenvironment. Second, oxidative stress is a key mediator of carbonyl stress, as glucose and lipids are transformed to reactive carbonyl compounds (RCOs), and excess RCOs are converted to advanced glycation end products (AGEs). Glyoxalase converts RCOs to glutathione (GSH), which is an antioxidant. Vitamin B6 detoxifies RCOs and AGEs, and its deficiency can impact the metabolism of many neurotransmitters, resulting in various neurotransmission deficits in brain function. Third, AGEs interact with the AGE receptor (RAGE), causing inflammation to rise. Increased neurotoxic metabolites as a result of inflammatory damage mediated by the Kynurenine pathway. Microglia are also activated, releasing inflammatory cytokines that cause dysregulation of neuronal circuits and neurodegeneration. Therefore, TRS, a neurological disorder, is caused by the combination of mentioned mechanisms. ROSs, reactive oxygen species; GLO, glyoxalase.

The above-mentioned discoveries on peripheral biomarkers of TRS provide new information on the neurobiological process of TRS. These neurobiological processes are interrelated, which result in abnormalities of neurological functioning, leading to the pathogenesis of TRS, as shown in Figure 2.

First, in schizophrenia, dysregulation of glutamate activity in neural circuits may be caused by an initial inflammatory injury mediated by the kynurenine pathway (Pedraz-Petrozzi et al., 2020), which is accompanied by a decrease in 5-HT synthesis but an increase in neurotoxic metabolites derived from tryptophan, such as 3-hydroxy-kynurenine (3HK), 3-hydroxy-anthranilic acid (3-OHAA), anthranilic acid and quinolinic acid (QA). Ultimately, this leads to neuronal circuit dysregulation and neurodegeneration (Möller et al., 2015). Second, in addition to factors involved in oxidative stress that are thought to cause monoamine changes, oxidative stress itself causes neurochemical and neuroanatomical changes (Nedic Erjavec et al., 2018). Furthermore, the increased peroxidation in TRS may indicate an aberrant fatty acid level in the synaptic membrane, resulting in neuronal dysfunction and associated abnormality in the microenvironment (Medina-Hernández et al., 2007). There’s also evidence that changes in the physicochemical milieu of lipids and fatty acids affect the affinity of membrane receptors for neurotransmitters including dopamine, GABAergic, and glutamate, which could be linked to adverse reactions to antipsychotic therapy in TRS patients (Fenton et al., 2000). The lipid peroxidation product 4-hydroxynonenal induced by oxidative stress can compromise the integrity of the blood-brain barrier and increase its permeability (Möller et al., 2015), resulting in elevated inflammatory response and systemic stress response (Nedic Erjavec et al., 2018). Third, in carbonyl stress, AGEs interact with the AGE receptor (RAGE), causing increased oxidative stress and inflammatory mediators (Kouidrat et al., 2015). Microglia get engaged when the brain is under psychological stress, releasing inflammatory cytokines that cause aberrant neurogenesis and neuronal degeneration (Katsuta et al., 2014). Because pyridoxal is a key coenzyme in the production of several neurotransmitters, including dopamine, serotonin, and GABA, VB6 deficiency can impact the metabolism of many neurotransmitters, resulting in various neurotransmission deficits in brain function (Itokawa et al., 2018; Toriumi et al., 2021).

## 6 Concluding remark, limitation and future perspective

### 6.1 Concluding remark

A series of peripheral biomarkers with high availability and feasibility have been demonstrated for TRS and were summarized in Table 1 – 3. First of all, molecular and genetic methods, such as pharmacogenomics, provide a means of predicting the molecular basis of treatment response. Furthermore, detection of peripheral immune system and stress biomarkers can be used to study the heterogeneity of psychotropic medication reactions in a simple and cost-effective manner. However, up to date, most biomarkers are still premature and no marker has been validated, for selectively and specificity in replication, or fully used in clinical practice on a consistent basis. A combination/panel of markers could be a more effective strategy for predicting and responding to treatment outcomes.

**TABLE 1:** Genetic biomarkers of treatment resistant schizophrenia.

Gene biomarkers	Risk genes for TRS	Subjects (Ethnic)	Measurement technique
Dopaminergic system			
COMT Escamilla et al., (2018)	Met/Met	TRS (*n* = 49), non-TRS (*n* = 8) Ultra-TRS (n = 33), Mexican	Whole blood, Flexigene DNA kit, TaqMan probes
COMT Inada et al., (2003)	L/L	Sch (*n* = 100), TRS (*n* = 20), Con (*n* = 201), Japanese	Whole blood, PCR, DNA silver staining kit
COMT Sagud et al., (2018)	rs4680 AA	TRS (*n* = 270), non-TRS (*n* = 661), Caucasian	Whole blood, TaqMan probes, Real time PCR
COMT Sagud et al., (2018)	rs4818 CC		
DRD1 Ota et al., (2012)	rs4532 (A/G, G/G)	TRS (*n* = 59), non-TRS (*n* = 65), European, African, American	Whole blood, Gentra Puregene kit, TaqMan probes,PCR
DRD2 Kondo et al., (2003)	Taq1A	Sch (*n* = 49)	Whole blood, Guanidinium isothiocyanate method, PCR
DRD2 Kondo et al., (2003)	-141cIns/Del		
DRD2 Escamilla et al., (2018)	A-241G		
DRD3 Escamilla et al., (2018)	Ser/Gly		
DRD3 Kohlrausch et al., (2008)	T/A/G/A/C haplotype	TRS (*n* = 121), non-TRS (*n* = 65), Brazilian	Peripheral blood leukocytes, PCR, Minisequencing assays, Mass spectrometry analysis
Serotonergic system			
TPH1 Anttila et al., (2007)	C/A	TRS (*n* = 51), non-TRS (*n* = 43), Con (*n* = 392), Finnish	Peripheral blood leukocytes, A kit and BioRobot M48 Workstation, TaqMan probes, PCR
HTR2A Herken et al., (2003)	T102C (T/C, T/T)	Sch (*n* = 141), Con (*n* = 79), Turkish	PCR
HTR2A Anttila et al., (2007)	T102C (C/C)		
HTR3B Kohlrausch et al., (2010)	-100_-102 del AAG	TRS (*n* = 101), non-TRS (*n* = 244), Japanese	Polymerase chain reaction-restriction fragment length polymorphism (PCR-RFLP), PCR
5-HTT Bilic et al., (2014)	5-HTTLPR (LaS)	TRS (*n* = 92), non-TRS (*n* = 81)	Whole blood, Salting out procedure, PCR
5-HTT Wang et al., (2007)	HTTVNTR (L-12)	non-TRS (*n* = 72), TRS (*n* = 57), Chinese	Peripheral blood lymphocytes, Capillary electrophoresis, PCR
DAT and SERT-in2 Bilic et al., (2014)	DAT 9/10, 9/9, 9/9 and 6/6 and SERT-in2ll, DAT 10/10 or 10/12 and SERT- in2ls or ss		
Glutamatergic/GABA System			
NMDA (GRIN2B) Chiu et al., (2003)	2664C/C	TRS (*n* = 193), Con (*n* = 176), Chinese	Peripheral white blood cells, PCR
NMDA (GRIN2B) Taylor et al., (2016)	rs1072388	TRS (*n* = 175), European	Venous blood samples, High salt method, TaqMan, PCR
GRM3 Bishop et al., (2011)	rs1989796	TRS (*n* = 95), Caucasian, African American, Asian	Whole blood, Salt precipitation method, Pyrosequencing^TM^ Technology
GRM3 Bishop et al., (2011)	rs1476455		
GRM3 Bishop et al., (2015)	rs1468412	Sch (*n* = 61), Con (*n* = 130), Caucasian, African American	Whole blood, Gentra Puregene extraction kit, Sequence-validated pyrosequencing, TaqMan assays, Sequenom MassARRAY platform
PI4KA and GRM3 Kaur et al., (2014)	rs165854 and rs1468412	Sch (*n* = 482), Con (*n* = 230), Indian	Whole blood, Modified salting-out procedure, SNaPshot^TM^, ddNTP primer extension kit
GAD1 Miyazawa et al., (2022)	rs3749034	TRS (*n* = 357), non - TRS (*n* = 682), Con (*n* = 508), Japanese	Whole blood, QI Amp DNA Blood Mini kit, High-throughput sequencing, TaqMan probe assays, Real-time PCR
GABBR2 Miyazawa et al., (2022)	rs10985765		
COMT and GAD1 Kogure et al., (2021)	rs4680 and rs3470934	TRS (*n* = 171), non -TRS (*n* = 592), Con (*n* = 94), Japanese	Blood sample, QIAamp DNA Blood Mini Kit, TaqMan probe assay, Real-time PCR
Cannabinoid system			
CB1 Hamdani et al., (2008)	rs1049353 GG	TRS (*n* = 59), non - TRS (*n* = 74, Con (*n* = 141), French	PCR
Others			
Cell signaling Moretti et al., (2018)	UFD1L	TRS (*n* = 78), non - TRS (*n* = 84, Con (*n* = 94)	Peripheral blood, TaqMan low-density array

COMT, Catechol-O-Methyltransferase; DRD1, dopamine receptor D1; DRD2, dopamine receptor D2; DRD3, dopamine receptor D3; TPH1, tryptophan hydroxylase 1; HTR2A, 5-hydroxytryptamine receptor 2A; HTR3B, 5-hydroxytryptamine receptor 3B; 5-HTT, serotonin transporter; DAT, dopamine transporter; SERT-in2, serotonin transporter-second intron; NMDA, N-methyl-D-aspartic acid; GRIN2B, glutamate ionotropic receptor NMDA type subunit 2B; GRM3, glutamate metabolism receptor 3; PI4KA, phosphatidylinositol 4-kinase alpha; GAD1, glutamate decarboxylase 1; GABBR2, gamma-aminobutyric acid type B receptor subunit 2; CB1-cannabinoid receptor type 1; Con, controls; Sch, schizophrenia; Ultra-TRS, patients who are resistant to clozapine.

**TABLE 2:** Inflammation and stress biomarkers of treatment resistant schizophrenia.

Biomarkers	Subjects (Ethnic)	Measurement technique	References
Inflammation			
IL-6↑	TRS (*n* = 66), non-TRS (*n* = 55), Con (*n* = 43), Iraq	Serum, ELISA	Al-Dujaili et al. (2021)
IL-6↑	TRS (*n* = 17), non-TRS (14), Con (*n* = 7), Italy	Plasma, ELISA	Maes, (2000)
IL-6↑	TRS (*n* = 15), non-TRS (12), Con (*n* = 15), Italy	Plasma, ELISA	Lin et al. (1998)
NK cells↑	TRS (*n* = 17), Con(*n* = 20), England	Venous blood, Multiparameter flow cytometry	Fernandez-Egea et al. (2016)
naive B cells↑			Fernandez-Egea et al. (2016)
CXCR5+ memory T cells↑			Fernandez-Egea et al. (2016)
classical monocytes↑			Fernandez-Egea et al. (2016)
DC ↓			Fernandez-Egea et al. (2016)
Tregs ↓			Fernandez-Egea et al. (2016)
CD4+memory T cells ↓			Fernandez-Egea et al. (2016)
NLR no change/↑	TRS (*n* = 22), non-TRS (29), UTRS (*n* = 105), Canada	Medical charts	Labonté et al. (2022)
PLR no change			Labonté et al. (2022)
DDK1↑			Al-Dujaili et al. (2021)
CC16↓			Lin et al. (1998)
CCL11↑			Al-Dujaili et al. (2021)
IL-12/IL-23p40↑	TRS (*n* = 39), non-TRS (156), UTRS (*n* = 15), French	Serum/plasma, MSD, ELISA	Leboyer et al. (2021)
IL-17A↑			Leboyer et al. (2021)
B2M↑			Leboyer et al. (2021)
IL-2↑	Sch(*n* = 78), Con(*n* = 30), China	Serum, ELISA	Zhang et al. (2004)
IL-8↑			Zhang et al. (2004)
IL-10↑			Al-Dujaili et al. (2021)
sTNF-R1↑	Sch(*n* = 54), Con(*n* = 118), Brazil	Serum, ELISA	Noto et al. (2015)
sTNF-R2↑			Noto et al. (2015)
MCP-1↑			Noto et al. (2015)
IL-1RA↑			Maes et al. (2000)
hsCRP↑	Sch(*n* = 82), Con(*n* = 50), Ethiopia	Blood sample, Turbidimetric and electrochemiluminescence immunoassay methods	Challa et al. (2021)
hsCRP no change	Sch(*n* = 390), Danish	Medical charts	Horsdal et al. (2017)
Short telomeres	TRS (*n* = 34), non-TRS (34), Con(*n* = 76), China	Blood samples, Southern blot analysis on the mean length of TRF	Yu et al. (2008)
Short telomeres	TRS (*n* = 21), non-TRS (20), Con(*n* = 36), Italy	Blood, Q-FISH	Squassina et al. (2020)
Stress			
MDA↑	TRS (*n* = 13), non-TRS (13), Con(*n* = 13), Mexican	Serum, Spectrophotometry	Medina-Hernández et al. (2007)
4-HNE↑			Medina-Hernández et al. (2007)
Pentosidine↑	Sch(*n* = 137), Con(*n* = 47), Japanese	Serum, ELISA, HPLC	Katsuta et al. (2014)
Pyridoxal↓			Katsuta et al. (2014)
EPA↓	131 patients	Whole blood, OmegaQuant, LLC	Messamore and McNamara, (2016)
DHA↓			Messamore and McNamara, (2016)
C22:5n3 ↓	Sch(*n* = 327), Con(*n* = 159), China	Blood samples, GC-MS	Li et al. (2022)
Omega 3 index↓			Li et al. (2022)

IL, interleukin; ELISA, enzyme-linked immunosorbent assay; NK cells, natural killer cells; Tregs, HLA-DR+ regulatory T-cells; DC, dendritic cells; NLR, neutrophil to lymphocyte ratio; PLR, platelet to lymphocyte ratio; DDK1, dickkopf-related protein; CCL11, eotaxin; MSD, Human V-Plex electrochemiluminescence assay; B2M, beta 2 microglobulin; sTNF-R1, soluble tumor necrosis factor receptor 1; sTNF-R2, soluble tumor necrosis factor receptor 2; MCP-1, monocyte chemotactic protein 1; hsCRP, C-reactive protein; TRF, terminal restriction fragment; Q-FISH, quantitative fluorescent *in situ* hybridization; MDA, malondialdehyde; 4-HNE, 4-hydroxynonenal; HPLC, high-performance liquid chromatography; EPA, eicosapentaenoic acid; DHA, docosahexaenoic acid; GC-MS, Gas Chromatography-Mass Spectrometry; Con, controls; Sch, schizophrenia; Ultra-TRS, patients who are resistant to clozapine.

**TABLE 3:** Genetic biomarkers of treatment resistant schizophrenia with clozapine treatment.

Gene biomarkers	Risk genes	Subjects (Ethnic)	Measurement technique
Dopaminergic system			
DRD1 Hwang et al., (2007)	rs265976A/C	TRS (*n* = 110), non-TRS (*n* = 122)	Blood samples, The high-salt method, 5′-exonuclease fluorescence assay, PCRs, TaqMan
		Caucasians, African-Americans	
DRD1 Potkin et al., (2003)	2,2 DRD1	TRS (*n* = 15), America	Blood samples, High-salt method, PCR
DRD2 Hwang et al., (2005)	TaqIB		
DRD2 Hwang et al., (2005)	rs1125394		
DRD2 Hwang et al., (2005)	TaqIA1		
DRD2 Potkin et al., (2003)	1,2 DRD2		
DRD3 Hwang et al., (2010)	rs2134655		
DRD3 Hwang et al., (2010)	rs2399504		
DRD3 Hwang et al., (2010)	rs1394016		
DRD3 M et al., (2020)	rs6280AG	TRS (*n* = 173), Ultra-TRS (*n* = 27), Indian	Blood sample; PCR; Sanger sequencing
DRD4 Hwang et al., (2012)	120-bp 1-copy		
DRD4 Hwang et al., (2012)	intron I (G)n 142-bp/140-bp		
DRD4 Hwang et al., (2012)	exon III 4R		
COMT Bosia et al., (2015)	val/met or met/met	TRS (*n* = 107), Italy	Whole blood, Illustra blood genomicPrep Midi Flow kit, PCR, MegaBACE™ 500 genetic analyzer
COMT and DRD4 Rajagopal et al., (2018)	val/met or met/met and 120-bp duplication	TRS (*n* = 93), Indian	Peripheral venous blood, RFLP
Serotonergic System			
HTR1A Bosia et al., (2015)	-1019 C/G		Whole blood, Illustra blood genomicPrep Midi Flow kit, PCR, MegaBACE™ 500 genetic analyzer
HTR2A Arranz et al., (1996)	Tyr452	TRS (*n* = 99), Ultra-TRS (*n* = 54), Con(n = 173), European, Caucasians	Whole blood, PCR
HTR2A Masellis, (1998)	Tyr 452	TRS (*n* = 97), Ultra-TRS (*n* = 88), Caucasians, African Americans, Asian	Blood samples, PCR-RFLP
HTR2A Arranz et al., (1998)	Tyr452	TRS (*n* = 181), Ultra-TRS (*n* = 93), Con(*n* = 178), British	PCR
HTR2A Arranz et al., (1998)	G-1438		
HTR3A Souza et al., (2010)	rs2276302	TRS (*n* = 140), Caucasian, African–American	Golden Gate assay, Taq Man platform
HTR3A Rajkumar et al., (2012)	rs2276302	TRS (*n* = 101), Indian	Peripheral venous blood, QIAamp DNA mini-kit, PCR
HTR3A Souza et al., (2010)	rs1062613		
HTR3A Rajkumar et al., (2012)	rs1062613		
HTR3A Souza et al., (2010)	rs1150226		
HTR3A Souza et al., (2010)	G-T-T haplotype		
5-HTT Kohlrausch et al., (2010)	HTTLPR/rs25531	TRS (*n* = 64), Ultra-TRS (*n* = 52), Southern Brazilian	Peripheral venous blood, PCR
Glutamatergic/GABA System			
GlyT1 Taylor et al., (2017)	rs16831558	TRS (*n* = 163), European	Peripheral venous blood, High salt method, Real-time PCR
Others			
CYP2C19 Rodrigues-Silva et al., (2020)	*1/*17	TRS (*n* = 63), Ultra-TRS (*n* = 45) Con(n = 137), Brazil	Whole blood, PCR
CYP3A4 Tóth et al., (2017)	Low expressers	TRS (*n* = 92), Hungarian	Peripheral blood, TaqMan probes, TaqMan Copy Number Assay, Real-time PC, LC-MS/MS
ABCB1 M et al., (2020)	rs1045642TT		
FKBP5 Mitjans et al., (2015)	rs1360780 TT	Sch (*n* = 591), British, Caucasians	Blood samples, Kompetitive allele specific PCR
NTRK2 Mitjans et al., (2015)	rs1778929 TT		
NTRK2 Mitjans et al., (2015)	rs10465180 CC		
ITIH3 Brandl et al., (2016)	rs2535629	Sch (*n* = 256), European, African-American	Blood, TaqMan Assay

DRD1, dopamine receptor D1; DRD2, dopamine receptor D2; DRD3, dopamine receptor D3; DRD4, dopamine receptor D4; COMT, Catechol-O-Methyltransferase; HTR1A, 5-hydroxytryptamine receptor 1A; HTR2A, 5-hydroxytryptamine receptor 2A; HTR3A, 5-hydroxytryptamine receptor 3A; 5-HTT, serotonin transporter; GlyT1, glycine transporter 1; ABCB1, adenosine triphosphate-binding cassette class B; FKBP5, the FK506 binding protein 5; NTRK2, neurotrophic tyrosine kinase receptor 2; ITIH3, Inter-alpha-trypsin inhibitor heavy chain H3; RFLP, restriction fragment length polymorphism; PCR, polymerase chain reaction; Con, controls; Sch, schizophrenia; Ultra-TRS, patients who are resistant to clozapine.

TRS may be a subtype of schizophrenia with distinct neurological foundations, based on the mounting results of antipsychotic responsiveness and biomarkers. However, compared with a portion of patients who fail to respond to initial treatment, many patients develop TRS as the disease progressed, which may be caused by the aggravation of neurobiological impairment caused by recurrence or long-term untreated psychosis (Takeuchi et al., 2019; Li et al., 2022). These two features cannot be disentangled according to the content of this review, the available data favors the combined effects of the two aspects.

### 6.2 Limitation

First, the biggest limitation in terms of the evidence was the difference in the definition of the TRS and the clozapine resistant patients, although they were defined according to the standards. The different study results may result from the treatment characteristics of the sample, such as treatment duration and single-drug or multi-drug treatment. Second, many cases need to be included in studies of genetic markers, especially rare CNVS, and thousands of carefully validated cases and matched controls need to be screened for unusual mutations. However, the number of large-scale prospective studies of genetic markers is relatively small. Third, on the one hand, some first-episode patients were resistant to treatment, while others responded to first-episode treatment and then did not respond to atypical antipsychotics. On the other hand, even response to clozapine varies after diagnosis of TRS. Patients were not stratified in these studies, and these differences resulted in a lack of replicated findings. Fourth, the use of specific prognostic biomarkers regarding the response to treatment in TRS should accordingly reflect the neurobiology of the specific phenotype. The identification of biomarkers reflecting treatment response may be impeded by the lack of established, accurate neurobiology of TRS. Finally, the CNS can immediately reflect pathological and biochemical abnormalities in patients with TRS because it is fundamentally a mental condition. However, due to the low cost and simple accessibility of peripheral biomarkers, as well as the high cost of neuroimaging technology and the impracticality of brain biopsy, researchers are very interested in peripheral biomarkers. Even while we recognize the importance of peripheral biomarkers, we may acknowledge that they are unable to correctly reflect the structural and functional alterations of the CNS. It is unclear if changes in peripheral biomarkers of TRS correspond to alterations in the CNS. The difficulty of biomarker identification lies in the development of a set of high-quality biomarker methods to ensure the measurement of real, accurate, sensitive and long-term stability.

### 6.3 Future perspective

When examining pertinent peripheral biomarkers of TRS, future studies should consider adhering to the TRS diagnostic consensus and recommendations. High sample sizes and reliable peripheral biomarker measuring and analysis techniques should be used to produce more precise and reproducible peripheral biomarkers. Stratification of schizophrenia patients in research is also critical. The subtypes of TRS may 1 day be distinguishable based on peripheral biomarkers and neurobiological mechanisms in addition to clinical factors (illness duration, number of episodes, predominance of negative symptoms, etc.). During the literature survey, we found that there is almost no animal model developed for TRS, which is an interesting topic that future researchers should strive to address. In a recent study, Rajagopal et al. recently used sub-chronic treatment of rodents with phencyclidine plus physiological stress to develop an antipsychotic-resistant model of schizophrenia in mice, which is worth digging up underlying mechanisms and clinical similarities (Rajagopal et al., 2022). Animal models for TRS, particularly those with cognitive impairment and negative symptoms, could provide novel insight into the pathophysiology, therapeutic strategy, and new pharmacological development for resistant individuals.
